# Comparison of Alternative Application Methods for Anti-*Varroa* Lithium Chloride Treatments

**DOI:** 10.3390/insects13070633

**Published:** 2022-07-15

**Authors:** Balázs Kolics, Éva Kolics, Kinga Mátyás, János Taller, András Specziár

**Affiliations:** 1 Festetics Bioinnovation Group, Institute of Genetics and Biotechnology, Georgikon Campus, Hungarian University of Agriculture and Life Sciences, H-8360 Keszthely, Hungary; bkolics@gmail.com (B.K.); petrovicsne.matyas.kinga.klara@uni-mate.hu (K.M.); taller.janos@uni-mate.hu (J.T.); 2 Balaton Limnological Research Institute, H-8237 Tihany, Hungary; specziar.andras@blki.hu

**Keywords:** *Apis mellifera*, application methods, lithium, trickling, *Varroa destructor*

## Abstract

**Simple Summary:**

The *Varroa* mite is one of the most dangerous pests of honey bee colonies, spreading many diseases, and its high infestation rate leads to the death of the colony. Due to its wide-scale distribution and lethal effect, this mite parasite threatens not only the honey industry but also the pollination of many plant species, including several cultivated plants. Therefore, an uncontrolled varroosis could threaten the human food supply as well as trigger biodiversity loss. Control options against *Varroa* are still inadequate; the mite could develop resistance to synthetic chemicals, whereas the efficiency of organic acid and essential oils-based treatments is very variable. Recently, lithium has been identified as a potential and effective anti-*Varroa* agent with both systematic and contact modes of action. Present experiments revealed that the efficiency of the lithium treatment depends greatly on the application method. The trickling method of application in repeated treatment and concentration of 500 mM provided very high, >>90%, efficiency. However, further investigations are required before the lithium chloride could be registered as a veterinary medicine in beekeeping practice.

**Abstract:**

Varroosis is one of the most dangerous threats to the bee industry but means of its treatment are still unsatisfactory. Lithium-based anti-*Varroa* treatments may provide an alternative, as this trace element can be a natural component of honey and is well tolerated by adult bees. However, it can be toxic to larvae and its use in beekeeping practice is not yet well understood. The present study aimed to investigate the efficacy of relevant application methods of acaricides used in beekeeping practice in brood-free conditions for lithium. Vaporisation proved to be an inefficient method of lithium treatment and killed only 9.9 ± 3.3% (mean ± SD) of mites in the hive. Lithium-impregnated paper strips showed moderate efficiency by killing 55.1 ± 26.2% of mites. The most effective way of applying lithium was the trickling method; different trickling treatments decreased the abundance of mites on average by 65 to 99.7%, depending on the applied dosage and the number of treatments. Repeated trickling treatments were more effective than single treatments, and they generally provided >90% efficiency. Experiments also proved that adding sugar to the trickling solution does not influence treatment efficiency. Thus, it is suggested that repeated and sugar-free trickling treatments with moderate lithium dosage could be the most rational methodology. Since lithium is not yet legalised in beekeeping practice, comprehensive studies are also needed to uncover the amount of lithium residue in bee products, depending on the treatment parameters.

## 1. Introduction

Ectoparasitic mite *Varroa destructor* [1] (*Acari*: *Varroidae*) is the biggest threat to both feral and managed honey bee colonies in its distribution area, which covers most of the world except Australia. It serves as a vector for various honey bee viruses, and it is recognised as the primary biotic cause of colony collapse in many regions of the world [2]. Left untreated, it can kill an entire colony within one or two years [3,4]; however, in areas of high bee density, this may occur within an apicultural season.

Means of control are restricted to only a few synthetic chemicals, implying the potential development of acaricide resistance [5,6]. As widely used alternatives, the application of hydrophilic organic acids (e.g., oxalic acid) is common because of their low risk of residues or accumulation in bee products [7,8,9]. However, they mostly require broodless conditions and may be largely dependent on climatic and in-hive conditions [10]. Thus, organic acids, as well as essential oils, provide variable control compared to synthetic acaricides. Extensive research has been carried out to fine-tune treatment protocols and to achieve a successful control [11,12,13,14,15,16,17,18,19,20,21].

Recently lithium was found to have varroacide properties, and it is highly effective in controlling the *Varroa destructor* population when administered to artificial swarms via feeding under broodless conditions [22,23]. In some treatments, 100% mite mortality was found with minor or no mortality of adult bees [22,24]; however, lithium may also harm bee larvae [22,25]. Environmental lithium can be a potential natural micronutrient component of foodstuffs of bees as well. Its quantity depends mainly on the soil’s lithium content [26,27]. Although lithium treatment based on the feeding method may leave traces in the honey, it appears that the lithium levels in honeys exposed to lithium treatment are below, or similar to, the trace-element levels in high-lithium honeys [28,29,30] or wax cappings [29], whereas it seems to leave brace combs and processed wax unaffected [28]. One of the main reasons for this may be that adult bees might be able to excrete the lithium in some way.

To perform anti-*Varroa* treatments on the superorganism of the hive, different approaches to administration are possible. However, not all of them may be relevant for a certain varroacide. In beekeeping practice, synthetic active substances are distributed by fogging, fumigation or aerosol formation (e.g., amitraz), impregnation onto cardboard or plastic strips to be inserted in between the combs (e.g., flumethrin, amitraz, coumaphos), or by trickling (e.g., coumaphos). The latter typically involves dripping a mixture of less than 50 drops over the bees between two combs (into the bee space). Although it is partly ingested by the bees and spread by colony trophyllaxis, the major part of the trickling solution is distributed externally by contact via comb cleaning and auto- and allogrooming. Alternatively, natural active substances can be applied by evaporation (e.g., formic acid), spraying (e.g., lactic acid) or sublimation, in the case of oxalic acid.

The potential use of lithium salts in beekeeping is approaching technology level application [24,31]. A method of feeding lithiated sugar syrup has emerged from the first in vitro experiments [22,29]. However, no other varroacide agent is known to date to be applied by feeding with winter food, mainly because of the potential risk of accumulation in bees and their products. It has been shown that lithium chloride (LiCl) has a significant contact effect as well against the mites [32], and can be exerted at the colony level by using lithiated paper strips or trickling lithiated sucrose solution in pre-wintering broodless colonies [31]. In addition, lithium chloride applied via trickling outperformed the efficiency of common oxalic acid treatment and showed an elongated effectiveness [31]. However, how the efficacy of lithium varies with the method of its application remains unknown.

Accordingly, the purpose of the present study was to compare the in situ anti-*Varroa* efficacy of alternative application methods of lithium. Specifically, first, we analysed how mite mortality varies between the trickling, vaporising and paper strip methods of application of lithium, and whether treatment efficacy is influenced by the infection rate (i.e., initial abundance of mites). Furthermore, since most of the previous studies have tested the efficacy of lithium admixed to a sugar syrup, a substance motivating the bees to ingest the lithium, we also examined whether the presence of the sugar has a valid effect on the efficacy of single and repeated trickling treatments.

## 2. Material and Methods

### 2.1. Experimental Set-Up

Experiments were carried out in situ by using full-sized colonies equipped in standard Hungarian hives (“Nagy Boczonádi NB18”). Hives were installed with a hygienic board to control the mite fall. Colonies were kept untreated for *Varroa* before the experiments, equalised to 10 combs (comb size: 40 cm× 34 cm), and rendered broodless via caging the queen.

In experiment I, we compared efficiencies of eight anti-*Varroa* LiCl treatment types. The trickling application method was tested in six different treatment setups with various LiCl concentrations in single and repeated treatments. Cold vaporising was carried out using VAT-1a device (Bee Research Institute, s.r.o, Dol, Czechia). Impregnated paper strips method of application was tested in single standard setups. Each treatment type was tested in five to 10 hives (Table 1). We also attempted to include the fumigation method of application of lithium in our experiments by using a Furetto-type equipment (Gremigni s.r.l., Firenze, Italy) and trying the following alternative carriers: petroleum (10%), glycerol (10%), paraffin oil (10%), and alcohol (10%). However, all of our preliminary attempts with fumigation failed (for more details see Results).

In experiment II, we compared the efficiency of 500 mM LiCl trickling treatments across three levels of sugar concentration of the medium (saturated sugar syrup, 1:1 mixture of water and saturated sugar syrup, and sugar-free water) and between single and repeated treatments. Each treatment type was tested in 9 to 10 hives (Table 1).

In terms of practical application, the amount of lithium should always be adjusted to the size of the comb and the colony. The volume of trickling is limited, since any excess solution dripped into the bee space may flow through the bees to the bottom board. Therefore, trickling volume was set to 40 mL for 10 combs, each with a total surface (both sides) of 2680 cm^2^ (comb equivalent: 1.5 mL/1000 cm^2^), and administered into bee space using an automatic veterinary syringe. An overview of the formulations, application doses and concentrations of trickling mixtures is given in Table 2 in comparison to their lithium equivalence (taking field tests of 80% varroacidal efficacy into account). In addition, a brief summary is also given in Table 2 on the dosages applied in the previous studies.

Each experiment was terminated by a lethal control treatment using two strips per colony of both Apivar (Véto-pharma, Palaiseau, France) and Check Mite+ (Bayer AG, Leverkusen, Germany) to assess the number of mites that resisted the LiCl treatment. Treatment efficiency was defined as the number of mites that died due to the LiCl treatment divided by the total number of mites in the experimental hive (mites died during the LiCl treatment + mites detected by the terminal control treatment).

### 2.2. Statistical Analysis

To evaluate variation in anti-*Varroa* efficiency between the tested treatment types, we used a general linear model (GLM) analysis. Explanatory factors were the treatment type in experiment I, the concentration of sugar (i.e., saturated, half of the saturated and zero) in the trickling solution, and the number of treatments (i.e., single or repeated) in experiment II. In order to take account of any density-dependent effect on treatment efficiency, the total number of mites was included in GLM analysis as a covariate in both experiment I and II.

First, a preliminary full factorial (test for homogeneity of slopes design) GLM was performed to explore whether the effect of the number of mites interacts with the effect of treatment type. In experiment I, preliminary GLM analysis revealed a significant interaction between the treatment type and the number of mites (d.f._error, effect_ = 7, 59, F = 3.35, *p* = 0.004). Therefore, we chose the separate-slopes design for the final GLM analysis, including the interaction of treatment type and the number of mites at the first position and treatment type (explanatory factor) at the second position. In experiment II, effect of the number of mites proved to be independent of both explanatory factors—concentration of sugar (d.f._error,effect_ = 2, 46, F = 0.15, *p* = 0.863) and number of treatments (d.f._error,effect_ = 1, 46, F = 2.36, *p* = 0.131)—as well as of their interaction (d.f._error,effect_ = 2, 46, F = 0.40, *p* = 0.674). Therefore, we chose the analysis of covariance (ANCOVA; homogeneous slope) design for the final GLM analysis, including the number of mites (as covariate) at the first position, the number of treatment types and the number of mites (explanatory factors), and, subsequently, their interaction. Visual inspection of frequency distribution and normal probability plots of model residuals and Levene’s tests supported the criteria of normality and homogeneity of variances of GLM analysis in both experiments.

Finally, we used the Tukey HSD post hoc test to identify pairwise differences in mean efficiency between treatment types. Statistical analyses were performed with the Statistica 8.0 software (http://www.statsoft.hu, accessed on 19 May 2021).

## 3. Results

**Figure 1:**
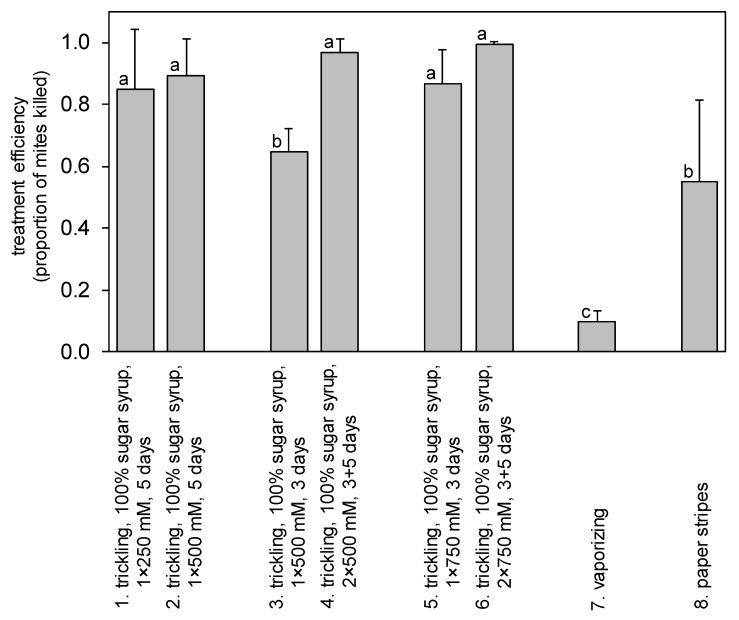
General linear model analysis considering the influence of the number of mites (covariate) on treatment efficiency via the separate-slopes design revealed significant variation in efficiency (mean ± SD) across eight anti-*Varroa* LiCl treatment types (for statistics, see Table 3). Plotted values marked with different letters are statistically different at *p* < 0.05 (Tukey HSD post hoc test).

In experiment I, the number of mites varied between 3 and 327 (mean ± SD: 54.3 ± 73.4) mites per hive and, as stated above, the influence of mite abundance on treatment efficiency varied across treatment types (Table S1, Figure S1a). The results of the fumigation method of application were excluded from the statistical evaluation because none of the tested carriers proved to be appropriate for treatment; the lithium salt was entrapped in the heat coil of the applicator. Separate-slopes design GLM model analysis identified significant variation in anti-*Varroa* efficiency across treatment types (Table 3 and Figure 1). Vaporising proved to be an inefficient method of lithium treatment and killed only 9.9 ± 3.3% (mean ± SD) of mites in the hive (Figure 1). Lithium-impregnated paper strips and trickling of 1 × 500 mM LiCl solution with 3 days observation period showed moderate mean efficiencies by killing 55.1 ± 26.2% and 64.8 ± 7.4% of the mites, respectively. The other five trickling treatment types showed similar and high mean efficiencies ranging between 85% and 99.7%.

**Figure 2:**
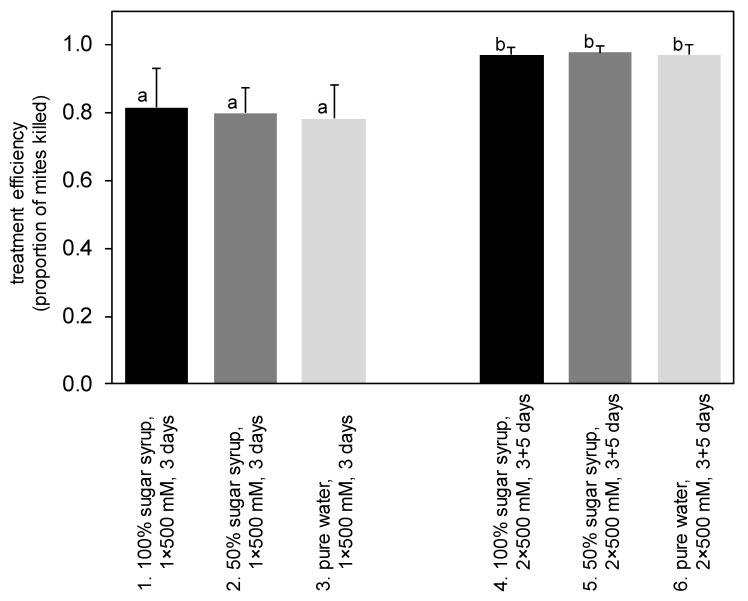
General linear model analysis considering the influence of the number of mites (covariate) on treatment efficiency via the ANCOVA (homogeneous slope) design revealed that efficiency (mean ± SD) of anti-*Varroa* LiCl treatments did not vary with the sugar concentration of the trickling solution. In contrast, repeated treatments proved to be more efficient than single treatments (for statistics, see Table 4). Plotted values marked with different letters are statistically different at *p* < 0.05 (Tukey HSD post hoc test).

In experiment II, the number of mites varied between 18 and 684 (245.0 ± 164.7) mites per hive, and the influence of mite abundance on treatment efficiency was homogeneous across treatment types; treatment efficiency tended to, but mostly did not significantly, decrease with increasing mite abundance (Table S1, Figure S1b). Analysis of covariance design GLM analysis revealed that the sugar concentration of the trickling solution had no effect on treatment efficiency (Table 4, Figure 2). On the other hand, repeated 500 mM LiCl trickling treatments proved to be significantly more efficient (97.3% ± 2.3%) than single treatments (79.7% ± 9.4%).

## 4. Discussion

Our experiments revealed that the efficiency of the recently proposed anti-*Varroa* lithium treatment depends greatly on the application method. Of the four commonly applied administration methods tested, one method proved to be unaccomplishable, two methods provided poor to moderate efficiency, and one method proved to be highly efficient.

### 4.1. Fumigation and Cold Vaporisation Methods

Although for some acaricide agents (e.g., amitraz, fluvalinate), application via fumigation may be applicable, our experiment showed that for lithium, fumigation with any of the tested carriers (petroleum, paraffin oil, glycerol, alcohol, glycerol + alcohol) was not suitable for the introduction of lithium chloride into the hive. The probable reason for this is that lithium chloride is insoluble in the carriers used. The water used as a solvent for the lithium evaporates during the process, causing the lithium salt to precipitate in the heating coil.

The introduction of lithium chloride solution into the hive via vaporisation as cold mist using VAT 1a was successful but showed low efficacy against the mite. A single treatment of 69.4 mg Li^+^ removed roughly 10% of the mite population. It is not comparable to amitraz fumigation, where administration of the active agent can reach a >90% efficacy [33], or oxalic acid evaporation, the most effective method of its administration, resulting in >90% efficacy [18], in a brood-free state.

### 4.2. Paper Strip Method

The impregnated strip method, where the active substance is incorporated into a plastic or paper strip, is a standard way of administration in defeating the *Varroa* mite based on a more prolonged exposure that may last for several reproduction cycles [34]. The paper strip method of the application relies mainly on the contact effect of lithium. Although it was revealed that it can result in a significant mite drop, this efficacy could be achieved by an increased dose compared to that used via trickling [32].

In the present experiments, administering the same amount of lithium chloride that was used in trickling (500 mM) resulted in only a moderate, 55.1%, efficacy in the paper strip method. Thus, we believe that the administration of lithium via paper strips is not a reliable alternative due to its lower effectiveness than trickling. In addition, the preparation of lithiated paper strips is more time-consuming than the trickling mixture. Moreover, it may lead to the generation of paper waste in the hive, which may increase the exposure to residues.

### 4.3. Trickling Method

Application of trickling mixture refers to dripping a small amount (<50 mL) of solution of the active ingredients, which may include sugar and other adjuvants. Here, the lid of the hive is removed, and the mixture is trickled onto the bees populating the bee’s space between the combs. Any droplets on the frames are sucked up, and all hive contaminants are cleaned up. Trickling as an appropriate application method for lithium was first proposed in 2020, suggesting that it is likely to have a bifold way of action, both systemic and contact [32]. It was found to be more effective than the routinely used oxalic acid in pre-wintering treatments [31].

Of the tested alternatives, the most effective way of administering lithium was the trickling method; different trickling treatments decreased the abundance of mites on average by 65 to 99.7%, depending on the applied dosage and the number of treatments. Higher concentrations were slightly more effective than 250 mM. However, the efficacy measured on day 8 of the experiment was significantly increased by repeated treatment on day 3 for all concentrations, indicating the need for repeated treatments. Although the lithium decontaminates relatively rapidly from the bees and their products [28,31], the tested concentration of 750 mM may pose an increased risk of residues without a statistically justifiable gain in the efficiency compared to the 500 mM concentration, especially in repeated treatment. Therefore, we consider that the optimal choice could be the 2 × 500 mM concentration lithium treatment via trickling, providing close-to-maximum effectiveness regardless of the number of mites present in the colony and with a lower risk of contamination than in more robust treatments. The 2 × 250 mM treatment could also be a suitable alternative in case of a lower estimated mite load. However, it is to note that the number of mites in a colony can only be roughly estimated based on the mite fall of the preceding period. In practice, the actual mite load can be revealed only at the onset of a brood-free stage in the pre-wintering period. Results of our experiment II suggest that the repetition of the trickling treatment itself may be more important than the applied concentration. Based on these findings, and because the lithium bears a prolonged effect [31] and only less than half of the mites survived the first trickling treatment in each trial, a 500 mM concentration primary treatment combined with a 250 mM concentration secondary treatment could also be a reasonable option.

Due to the high hygroscopicity of lithium chloride powder, it is practical to make a stock solution to prepare further dilutions, e.g., with sugar syrup. The sugar solution was used in most trickling treatments in the previous studies [31,32], with the presumption that it may facilitate the spread of lithium within the hive by representing an attractive food for the bees. However, our results show that the sugar content of the trickling solution does not affect the effectiveness of the treatment. Therefore, the preparation of a trickling solution with water may be more useful in practice than a dilute sugar solution because (i) it has a longer shelf life and (ii) making a trickling solution is possible without preparing a stock solution. Further studies are needed, however, to determine whether the amount of chemical residue in bees and their products is affected by the sugar content of the trickling solution, primarily because this factor could influence the likelihood that the agent is ingested by bees.

## 5. Conclusions

Our study aimed to identify the most effective method according to the modes of application of the currently used acaricides for lithium. Of the methods compared, the trickling method—facilitating both the systematic and contact type of anti-*Varroa* action of the lithium—was found to be the most appropriate method of application, with its efficacy comparable to traditionally used anti-*Varroa* agents. To achieve maximum efficiency, two consecutive treatments could be needed. The trickling method can be easily adapted to any type of hive by recalculating the solution per bee space according to the comb size and can be applied at most seasons of the year.

Although lithium shows high efficacy against mites and appears to be a promising alternative in brood-free conditions, it is important to note that it is currently not yet registered as a veterinary product in most countries. We aimed to reveal the appropriate application methods for lithium in apiculture if it ever becomes a recognised alternative to anti-*Varroa* treatments. Until potential risks of lithium are clarified in terms of chemical residues in harvested honey, a precautionary approach to lithium in beekeeping is recommended.

## Figures and Tables

**Table 1:** Specifications of the tested anti-*Varroa* LiCl treatment types.

	Treatment 1				Treatment 2				
Treatment Type	Method	Concentration LiCl × 1H_2_O	Li+ Dosage (mg)	Observation Period	Method	Concentration LiCl × 1H_2_O	Li+ Dosage (mg)	Observation Period	Number of Hives Treated
Experiment I									
1.	trickling, 100% sugar syrup	250 mM	69.4	5 days	-	-		-	10
2.	trickling, 100% sugar syrup	500 mM	138.8	5 days	-	-		-	10
3.	trickling, 100% sugar syrup	500 mM	138.8	3 days	-	-		-	10
4.	trickling, 100% sugar syrup	500 mM	138.8	3 days	trickling	500 mM	138.8	5 days	10
5.	trickling, 100% sugar syrup	750 mM	208.2	3 days	-	-		-	10
6.	trickling, 100% sugar syrup	750 mM	208.2	3 days	trickling	750 mM	208.2	5 days	10
7.	vaporising	5.5 M	69.4	8 days	-	-		-	5
8.	paper strips	5.5 M	138.8	8 days	-	-		-	10
Experiment II									
1.	trickling, 100% sugar syrup	500 mM	138.8	3 days	-	-		-	9
2.	trickling, 50% sugar syrup	500 mM	138.8	3 days	-	-		-	10
3.	trickling, pure water	500 mM	138.8	3 days	-	-		-	10
4.	trickling, 100% sugar syrup	500 mM	138.8	3 days	trickling	500 mM	138.8	5 days	9
5.	trickling, 50% sugar syrup	500 mM	138.8	3 days	trickling	500 mM	138.8	5 days	10
6.	trickling, pure water	500 mM	138.8	3 days	trickling	500 mM	138.8	5 days	10

**Table 2:** (**a**) Preparation of the trickling solution of different concentrations; (**b**) preparation of the trickling solution from stock solution; and (**c**) concentration of trickling solutions in comparison with previous studies.

(a)						
Preparation of trickling solution directly from different compounds		250 mM solution		500 mM solution		750 mM solution
Lithium chloride monohydrate (LiCl × H_2_O)		15.10 g L^−1^		30.20 g L^−1^		45.29 g L^−1^
Lithium chloride anhydrate (LiCl)		10.60 g L^−1^		21.20 g L^−1^		31.79 g L^−1^
(**b**)						
	Preparation of stock solution			Preparation of trickling solution from the stock		
	agent used (g)	final volume (mL)	final concentration (M)	stock solution (mL)	final volume (mL)	final concentration (mM)
LiCl anhydrate	500	2137	5.5	45.3	1000	250
LiCl monohydrate	500	1500	5.5	45.3	1000	250
(**c**)						
		Concentration	Single volume/colony	Way of administration	Single dose/ colony Li^+^ basis (mg)	Reference
Lithium chloride		25 mM	ad libitum	feeding sugar syrup	-	Ziegelmann et al., 2018 [22] Ziegelmann et al., 2019 [23]
Lithium chloride		50 mM	ad libitum	feeding sugar syrup	-	Ziegelmann et al., 2018 [22]
Lithium chloride		50 mM	ad libitum	feeding sugar dough	-	Ziegelmann et al., 2019 [23]
Lithium chloride		25 mM	1000 mL	feeding sugar syrup	173.5	Kolics et al., 2019 [30]
						Presern et al., 2020 [25], Kolics et al., 2021a [31]
Lithium chloride		250 mM	40 mL	trickling	69.4	Kolics et al., 2021b [31], Kolics et al., 2020 [32], present study
Lithium chloride		500 mM	40 mL	trickling	138.8	present study
Lithium chloride		750 mM	40 mL	trickling	208.2	present study
Lithium citrate		5 mM	1000 mL	feeding sugar syrup	101	Stanimirovic et al., 2022 [29]
Lithium citrate		10 mM	1000 mL	feeding sugar syrup	202	Stanimirovic et al., 2022 [29]
Lithium citrate		15 mM	1000 mL	feeding sugar syrup	302.9	Stanimirovic et al., 2022 [29]
Lithium citrate		20 mM	1000 mL	feeding sugar syrup	403.9	Stanimirovic et al., 2022 [29]
Lithium citrate		25 mM	1000 mL	feeding sugar syrup	504.9	Stanimirovic et al., 2022 [29]

**Table 3:** General linear model analysis considering the influence of the number of mites (covariate) on treatment efficiency via the separate-slopes design revealed significant variation in efficiency across eight tested anti-*Varroa* LiCl treatment types. Results of Tukey HSD post hoc tests are shown in Figure 1.

	Effect				Overall Model		
	d.f._error effect_	F	*p*	R^2^_adj._	d.f._model, residual_	F	*p*
Treatment × number of mites	8, 59	7.2	<0.001				
Treatment	7, 59	21.5	<0.001				
Overall model				0.850	15, 59	28.9	<0.001

**Table 4:** General linear model analysis considering the influence of the number of mites (covariate) on treatment efficiency via the ANCOVA (homogeneous slope) design revealed that the efficiency of anti-*Varroa* LiCl treatments did not vary with the sugar concentration of the trickling solution, whereas it differed between single and repeated treatments. Results of Tukey HSD post hoc tests are shown in Figure 2.

	Effect			Overall Model			
	d.f._error, effect_	F	*p*	R^2^_adj._	d.f._model, residual_	F	*p*
Number of mites	1, 57	5.6	0.021				
Sugar concentration	2, 57	0.8	0.469				
Number of treatments	1, 57	96.2	<0.001				
Sugar × number of treatments	2, 57	0.3	0.762				
Overall model				0.632	6, 51	17.3	<0.001

## Data Availability

Data is available at the Festetics Bioinnovation Group, Institute of Genetics and Biotechnology, Georgikon Campus, MATE.

## Supplementary Materials

The following supporting information can be downloaded at: https://www.mdpi.com/article/10.3390/insects13070633/s1, Figure S1: Relationship between the efficiencies of different anti-*Varroa* LiCl treatments and the number of mites in experiment I. (a) and experiment II. (b). Regression statistics are presented in Table S1.; Table S1: Linear regression analysis revealed that the efficiency of anti-*Varroa* LiCl treatments decreased or not varied with increasing mite abundance in experiment I. Still, efficiencies of treatments mostly tended to increase with increasing mite abundance in Experiment II. Relationships are visualised in Figure S1.
