# Supplementary material for: Comparison of Alternative Application Methods for Anti-Varroa Lithium Chloride Treatments
Source: Insects. 2022 Jul 15;13(7):633. doi: 10.3390/insects13070633 (PMC9318583; doi:10.3390/insects13070633)
Supplement: Supplementary file 1 [file insects-13-00633-s001.zip › insects-1785771-supplementary.pdf]

Table S1. Linear regression analysis revealed that the efficiency of anti-*Varroa* LiCl treatments decreased or not varied with increasing mite abundance in experiment I. Still, efficiencies of treatments mostly tended to increase with increasing mite abundance in Experiment II. Relationships are visualised in Figure S1.

	Intercept			Slope			Model fit			
	mean (95% CI)	t	P	mean (95% CI)	t	P	$R^2_{adj.}$	d.f.	F	P
Experiment I.										
**1. trickling, 100% sugar syrup, 1×250 mM LiCl, 5 days**	**0.964 (0.851 - 1.076)**	**20.0**	**<0.001**	**-0.0024 (-0.0039 − -0.0010)**	**-3.8**	**0.005**	**0.602**	**1, 8**	**14.6**	**0.005**
**2. trickling, 100% sugar syrup, 1×500 mM LiCl, 5 days**	**0.981 (0.933 - 1.029)**	**47.3**	**<0.001**	**-0.0013 (-0.0018 − 0.0008)**	**-6.5**	**<0.001**	**0.819**	**1, 8**	**41.8**	**<0.001**
3. trickling, 100% sugar syrup, 1×500 mM LiCl, 3 days	0.647 (0.564 - 0.730)	17.9	<0.001	0.0000 (-0.0022 − 0.0022)	0.0	0.974	0.000	1, 8	0.0	0.974
4. trickling, 100% sugar syrup, 2×500 mM LiCl, 3+5 days	0.987 (0.937 - 1.036)	48.0	<0.001	-0.0007 (-0.0020 − 0.006)	-1.3	0.243	0.062	1, 8	1.6	0.243
5. trickling, 100% sugar syrup, 1×750 mM LiCl, 3 days	0.879 (0.770 - 0.989)	18.5	<0.001	-0.0002 (-0.0013 − 0.0009)	-0.4	0.699	0.000	1, 8	0.2	0.699
6. trickling, 100% sugar syrup, 2×750 mM LiCl, 3+5 days	1.000 (0.995 - 1.005)	458.7	<0.001	-0.0001 (-0.0001 − 0.0000)	-2.3	0.053	0.315	1, 8	5.1	0.059
7. vaporizing	0.110 (-0.001 - 0.222)	3.1	0.052	-0.0001 (-0.0001 − 0.0001)	-0.4	0.744	0.000	1, 5	0.1	0.744
**8. paper stripes**	**0.629 (0.467 - 0.790)**	**9.0**	**<0.001**	**-0.0018 (-0.0033 − -0.0002)**	**-2.7**	**0.028**	**0.407**	**1, 8**	**7.2**	**0.028**
Experiment II.										
1. trickling, 100% sugar syrup, 1×500 mM LiCl, 3 days	0.741 (0.529 - 0.952)	8.3	<0.001	0.00023 (-0.00036 - 0.00082)	0.9	0.389	0.020	7	0.8	0.389
**2. trickling, 50% sugar syrup, 1×500 mM LiCl, 3 days**	**0.742 (0.672 - 0.811)**	**24.5**	**<0.001**	**0.00036 (0.00001 - 0.00072)**	**2.4**	**0.043**	**0.345**	**8**	**5.7**	**0.043**
3. trickling, pure water, 1×500 mM LiCl, 3 days	0.743 (0.598 - 0.887)	11.8	<0.001	0.00015 (-0.00032 - 0.00061)	0.7	0.492	0.057	8	0.5	0.492
4. trickling, 100% sugar syrup, 2×500 mM LiCl, 3+5 days	0.962 (0.921 - 1.003)	55.2	<0.001	0.00003 (-0.00009 - 0.00014)	0.6	0.563	0.086	7	0.4	0.563
5. trickling, 50% sugar syrup, 2×500 mM LiCl, 3+5 days	0.972 (0.949 - 0.995)	96.6	<0.001	0.00004 (-0.00008 - 0.00015)	0.7	0.488	0.055	8	0.5	0.488
6. trickling, pure water, 2×500 mM LiCl, 3+5 days	0.946 (0.91 - 0.981)	61.2	<0.001	0.0009 (-0.00002 - 0.00021)	1.9	0.099	0.217	8	3.5	0.099

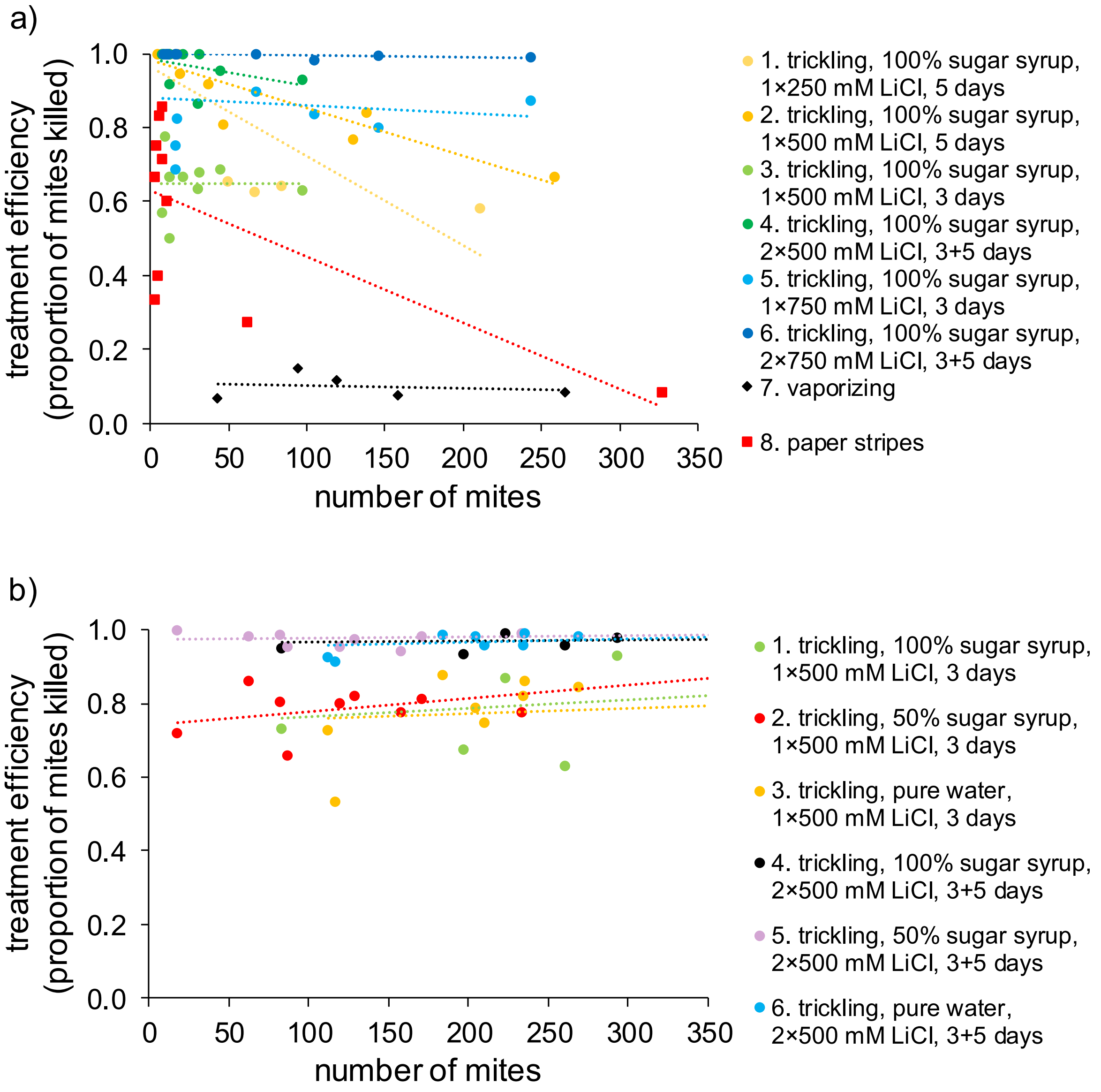

Figure S1. Relationship between the efficiencies of different anti-*Varroa* LiCl treatments and the number of mites in experiment I. (a) and experiment II. (b). Regression statistics are presented in Table S1.
